# Insightful Imagery is Related to Working Memory Updating

**DOI:** 10.3389/fpsyg.2016.00137

**Published:** 2016-02-29

**Authors:** Edward Nęcka, Piotr Żak, Aleksandra Gruszka

**Affiliations:** ^1^Institute of Psychology, Jagiellonian University in KrakowKrakow, Poland; ^2^The University of Social Sciences and HumanitiesWarsaw, Poland

**Keywords:** creativity, insight, imagery, working memory, n-back

## Abstract

Available body of evidence concerning the relationship between insight problem solving and working memory (WM) is ambiguous. Several authors propose that restructuring of the problem representation requires controlled search processes, which needs planning and involvement of WM. Other researchers suggest that the restructuring is achieved through the automatic spread of activation in long-term memory, assigning a limited role to WM capacity. In the present study we examined the correlations between insight problem solving performance and measures of WM updating function (n-back task), including general intelligence (as measured by Raven’s Advanced Progressive Matrices). The results revealed that updating function shared up to 30% of variance with the insight problem task performance, even when the influence of general mental ability was controlled for. These results suggest that insight problem solving is constrained by individual ability to update the content of WM.

## Introduction

In this paper we suggest that insightful problem solving depends on the efficiency of working memory (WM) functioning. In order to justify such a hypothesis, we first review the theoretical models of insight as an essential phenomenon in creativity and problem solving. Then, we summarize the existing empirical evidence, which is ambiguous in reference to the role of WM in insightful problem solving. We discuss possible sources of these ambiguities. Finally, we report the results of an empirical study, which suggests that the ability to solve insight problems may share as much as 30% of common variance with the updating function of WM, assessed with the n-back task.

Creativity is usually investigated within the framework of creative cognition (Finke et al., 1992; Smith et al., 1995). This approach assumes that creative outcomes occur through the application of “regular” cognitive processes, organized in a specific way so that their final output meets the criteria of novelty or originality. Numerous cognitive processes have been investigated within the creative cognition approach, including imagery (Finke et al., 1992; Ward, 1994; Finke, 1996; Palmiero et al., 2011), attention (Nęcka, 1999), executive control (Beaty et al., 2014; Benedek et al., 2014), or associative memory (Benedek and Neubauer, 2013). In the present study we focus on the relationship of creativity with a specific module of human memory called WM (Baddeley and Hitch, 1974; Baddeley, 2002). We also focus on a specific aspect of creativity, namely insight (Scheerer, 1963).

Historically, the psychology of creativity has been developing within two traditions. The first one descends from the Gestalt theory of productive thinking (Wertheimer, 1945), according to which creativity necessitates restructuring of the original mental representation of the problem (Ohlsson, 1984a,b). In empirical studies, the proponents of this tradition typically use the so-called insight tasks, which have one well-defined solution but involve an element of a “mental trap” that must be eliminated or overcome (Weisberg, 1995). For instance, in order to solve the task: “Create four equilateral triangles with six matchsticks,” one has to imagine a pyramid in the three-dimensional space rather than attempting in vain to remain on the one-dimensional plane. Similarly, the matchstick task, which requires rearrangement of just one matchstick in order to obtain a valid equation expressed in Roman numeric symbols (e.g., nonsensical III + III = III can be rearranged into tautological but valid III = III = III, DeCaro et al., 2015), needs imaginary transformation of the symbol “+” into “=”. Regardless of their being verbal or non-verbal in nature, insight problems usually need active engagement of mental imagery, as the above-mentioned problems show clearly.

The second tradition of creativity studies descends from the theory of divergent thinking (Guilford, 1950, 1967; Wallach and Kogan, 1965), according to which creativity is connected with unconstrained search for solutions in many different directions. Ideas generated in the process of divergent thinking differ in their value, originality, or simply appropriateness to the problem at hand. Therefore, they must be selected and elaborated upon in order to find out the really creative ones. The well-known rule of brainstorming, stating that “quantity breeds quality” (Osborn, 1953; Parnes and Meadow, 1959), also known as the “equal-odds rule” (Simonton, 1997; Jung et al., 2015), seems to be compatible with this approach. The proponents of this tradition typically investigate creativity using the so-called divergent problems, which have many acceptable solutions and need fluency of thinking rather than restructuring. For instance, the question of unusual uses of a brick (i.e., “How many unusual and uncommon uses can you come up with for a *brick*?”, Guilford, 1967) does not have any “correct” solution nor does it need any type of restructuring. It just relies on the problem solver’s ability to think in a fluent and flexible way.

In mature real-life creativity both insight-based and divergent thinking processes are probably intertwined but in laboratory studies one has to decide on a particular approach in order to adopt the appropriate research paradigm. In this study, we decided to investigate the cognitive correlates of insight.

Insight is defined as a sudden realization that the hitherto unresolved and seemingly very difficult problem can be easily solved if it is perceived from a new perspective (Scheerer, 1963; Dominowski and Dallob, 1995; Ansburg, 2000; Chu and MacGregor, 2011). Such a change of perspective cannot be achieved through conscious effort and planning, so suddenness is its typical attribute (Davidson, 1995). Subjectively, insight is usually accompanied with the “Aha!” experience. It is preceded by many unsuccessful attempts to resolve the problem in the routine way. Since the routine approaches do not work, the experience of impasse is inevitable. The period after the impasse is called “incubational break,” during which no apparent mental activity is observed, both objectively and in introspection (Sio and Ormerod, 2009). Then, an entirely new idea appears in one’s mind, as if coming from “nowhere.” This pattern of creative problem solving can be found in many instances of scientific discoveries (Simonton, 1988; Dunbar, 1995; Csikszentmihalyi, 1996). Being a pivotal moment of creativity, insight has been extensively studied by psychologists in hope of revealing its cognitive mechanisms.

In contemporary models, the cognitive machinery of insight amounts to restructuring (Ohlsson, 1984a,b; Ash and Wiley, 2006). This phenomenon can be described as rearrangement of elements of the problem’s cognitive representation. Suddenly, elements of the cognitive representation that up to now seemed crucial appear unimportant, whereas those that seemed irrelevant gain utmost importance. In other words, a new pattern, or Gestalt (Wertheimer, 1945), comes to one’s mind, thus suggesting a new and productive way of thinking. What causes such a rearrangement is not clear yet. The theory of selectivity (Davidson, 1995) claims that the problem solver’s ability to selectively encode, compare, and combine the elements of cognitive representation leads to restructuring. It is not clear what is the nature and origin of this ability to think selectively, although there are suppositions that it might be the matter of conscious and controlled efforts to process information in the selective way (e.g., Ash and Wiley, 2006). According to Simon (1977), insight occurs when irrelevant aspects of the problem are selectively forgotten, thus making the complex and difficult problem familiar and simplified enough (see also: Simon et al., 1981; Langley and Jones, 1988). Another group of theories underscores the role of opportunistic assimilation of information (e.g., Seifert et al., 1995). Accidental stimuli appearing in the environment are opportunistically assimilated with the original mental representation, thus producing a new, rearranged pattern of relationships between separate elements of the problem representation. They may also suggest a novel solution thanks to the analogical transfer of knowledge (Ormerod et al., 2006).

Taking into account the cognitive characteristics of insight, we hypothesize that its occurrence should depend significantly on WM processes. WM is responsible for active maintenance of information relevant to the problem at hand. It is also believed to enable active manipulation with the elements of the problem’s mental representation (Baddeley and Hitch, 1974; Cowan, 2001, 2010; Baddeley, 2002; Engle, 2002; Unsworth and Engle, 2007a). Active maintenance is possible thanks to the modules called articulatory loop and inner scribe, connected with verbal and non-verbal material respectively, whereas manipulation with the problem’s elements is ascribed to the central executive (Baddeley, 2002). If insight amounts to restructuring of mental representation of the problem, its cognitive machinery must rely greatly on the WM mechanisms. Restructuring probably starts with the decomposition of distinct elements of the problem’s structure. Then, it requires the maintenance of these elements in active WM in order to use them in multiple attempts to build up a new structure. Finally, new structures are constantly built and rebuilt, which is a process resembling the creation of temporary bindings among the elements kept in the primary memory (Oberauer et al., 2007; Oberauer, 2009). New structures typically utilize elements that were formerly ignored as ostensibly irrelevant or redundant. Since the problem solver does not realize from the very beginning which elements are relevant or not relevant, he/she must keep in active memory as many elements as possible. So, capacious WM should increase the likelihood of the occurrence of insight. Moreover, formerly “irrelevant” chunks of information are probably kept in less active parts of WM, i.e., outside of the focus of attention (Cowan, 2001), or even in the LTM store, that is, in the inactive state of mental representation. If so, they must be activated and transferred to the focus of attention in order to be fully prepared for immediate utilization in thinking processes. Hence, executive aspects of WM, also called “controlled attention” (Engle et al., 1999a; Engle, 2002; Unsworth and Engle, 2007a), should contribute to the likelihood of insight and its quality, too. To sum up, the cognitive analysis of insight leads us to the hypothesis that insightful thinking, or insightful imagery, is almost synonymous to WM processes, as they are conceptualized in the most influential theoretical models (e.g., Engle et al., 1999a; Cowan, 2001, 2010; Baddeley, 2002).

Available empirical evidence is quite ambiguous in this respect. On the one hand, there are studies reporting at least moderate correlations between batteries of insight tasks and various measures of WM. For instance, Murray and Byrne (2005) used the battery of eight insight tasks and obtained the correlation of *r* = 0.39 with backward-digit task and *r* = 0.51 with the span task. Gilhooly and Fioratou (2009) also report positive correlations between insight problems and both verbal and non-verbal WM span tasks (*r* coefficients ranged between 0.27 and 0.38), although correlations with non-insight problems were approximately at the same level. Interestingly, this study demonstrated that executive control, in contrast to WM, entered into much weaker associations with insight problem solving, a result reported in other studies, too (Paulewicz et al., 2007). De Dreu et al. (2012) showed that WM contributed to creative outputs in general, and correct solutions to insight problems (i.e., Remote Association Rest’s items) in particular. Yeh et al. (2014) demonstrated that WM capacity helped to solve insight problems in interaction with attention and eye movements. Arguments for the essential role of WM capacity in insightful problem solving can also be found in other studies (e.g., Chein et al., 2010; Chein and Weisberg, 2014).

On the other hand, many studies report the results that are not compatible with our hypothesis. For example, Gilhooly and Murphy (2005), using a painstakingly selected set of “pure” insight problems, did not observe any significant correlation with the backward-digit tasks. They reported a weak correlation with the span task (*r* = 0.23) but, notably, analytical problems, which did not require any insight, correlated with the span task at the same level. Other studies also support the supposition that WM capacity may predict analytical rather than creative thinking processes. For instance, Lavric et al. (2000) asked people to solve insight and analytical problems while simultaneously counting tones generated by the computer. They found that engagement of WM through counting did not affect insight problem solving, contrary to analytical problem solving. DeCaro et al. (2015; see also: Van Stockum and DeCaro, 2013) go as far as to argue that “WM capacity constraints insight” because it leads people to employ complex thinking strategies, whereas insight, according to them, needs remote associations rather than resource-dependent complex thinking. It is also argued that insight cannot be reached through deliberate planning (Chein et al., 2010), which is one of the vital functions ascribed to WM (Gilhooly, 2005).

A question arises, what are the causes of such inconsistencies in the available literature. The answer lies probably in the methodological weaknesses, which are notorious in the insight problem solving studies (Gilhooly and Murphy, 2005). To begin with, in many studies the authors used just one category of problems, such as the tasks which require rearrangement of just one matchstick in order to obtain a valid equation expressed in Roman numeric symbols (DeCaro et al., 2015), remote association tasks (De Dreu et al., 2012), or compound remote association tasks (Chein and Weisberg, 2014). It seems that, for psychometric reasons, more diversified batteries of insight tasks should be applied. Additionally, the batteries of insight tasks are sometimes very short, consisting of one (Chein et al., 2010), four (DeCaro et al., 2015), six (Ash and Wiley, 2006), eight (Murray and Byrne, 2005), or ten (Paulewicz et al., 2007) items. It seems that psychometric properties of short batteries can be criticized, especially if a particular study is designed according to the individual differences approach. Purity of the insight task batteries may also be questioned, as they happen to involve the tasks that can be solved analytically as well (Weisberg, 1995; Gilhooly and Murphy, 2005). Finally, familiarity of insight tasks is usually not controlled, although prior knowledge makes them entirely non-problematic. As to WM measures used in the insight studies, they tend to be rather complex and multifaceted, thus excluding the opportunity to investigate specific cognitive processes involved. Moreover, complex WM measures, such as the span tasks, may be interpreted as proxies for intelligence tests, since their results are usually strongly correlated with intelligence tests’ results. Finally, the span tasks refer to the “mnemonic” aspects of WM, performed by the articulatory loop or the inner scribe, rather than to its “processing” aspects, carried out by the central executive. We have not been able to find a study of insight in which some specific function of WM would be investigated, such as the function of updating (Morris and Jones, 1990).

Updating consists in constant rearrangements of the temporal order among the items kept in the primary memory. Participants are asked, for instance, to recall the last *n* items of a long list of elements (Morris and Jones, 1990) or to decide whether the current element of the running list has been already presented *n* items back (McElree, 2001). In order to do such tasks, one has to revise the temporal order of the list of elements, since the element presented two items back is going to take the position of three items back, and then four items back, and so forth. In other words, one has to keep in active memory as many elements as possible but also rearrange their temporal order. The function of updating predicts individual differences in complex cognitive skills (Miyake et al., 2000; Friedman et al., 2006; Ecker et al., 2010), although insight problem solving has not been studied extensively from this perspective. Moreover, the processes hypothesized to operate during updating, such as retrieval, transformation, and substitution of elements (Ecker et al., 2010), or binding (Wilhelm et al., 2013), resemble the processes involved in insightful restructuring. It is therefore interesting to investigate possible relationships between the efficiency of the function of updating and the ability to solve insight problems.

In our study, we selected a quite large battery of insight problems. We also focused on the function of WM updating rather than using a complex span measure of WM capacity. Finally, we decided to assess the general intelligence level, so as to be able to look for potential relationships between WM capacity and insight problem solving when general intelligence is controlled for. Apart from being determined by WM processes, intelligence is by definition the general ability to solve problems, particularly the complex and abstract ones. Therefore, investigation of the relationships between WM and insightful problem solving needs checking for possible correlations confounded by the general intelligence.

## Materials and Methods

### Participants

We investigated 91 male volunteers. Their age ranged between 18 and 26, *M* = 21.36, *SD* = 3.62. They were high school and university students outside of psychology department. We recruited them through advertisements disseminated at their residences. One participant’s data were not saved on the disk, another one resigned before completing the tests. Both had to be excluded, leaving 89 persons in the final sample.

### Ethical Statement

The committee for ethics in studies involving human participants, assigned by the Department of Psychology, Jagiellonian University in Krakow, approved this study on the basis of extended description of methods, materials, and procedure. According to the Helsinki declaration, participants signed written informed consent forms.

### Tests and Materials

#### Insight Tasks

In order to investigate the participants’ ability to solve insight problems, we selected a set of 31 tasks that are believed to require insightful skills. In the beginning, we gathered all available tasks reported in the literature, particularly in the works by Metcalfe and Wiebe (1987), Schooler et al. (1993), Dominowski and Dallob (1995), Gick and Lockhart (1995), Isaak and Just (1995), Weisberg (1995), and Gilhooly and Murphy (2005). We also took into account a 68-item test used in the study on training insight problem solving (Dow and Mayer, 2004). Some tasks overlapped across the analyzed studies. Others raised doubts concerning their theoretical validity. Therefore we decided to reduce the number of items on the basis of three criteria. First, we excluded the items that failed empirical verification as insight tasks (Dow and Mayer, 2004; Gilhooly and Murphy, 2005). Second, we excluded the items that have been discussed in the literature as questionable concerning their insight nature. Third, we excluded the items that were culturally specific, for instance, the ones that could be solved only if a person would possess specific knowledge concerning culture, tradition, or religion.

In such a way, we obtained 66 items that have been subjected to the procedure suggested by Davidson (1995). We asked 52 judges to solve and evaluate these 66 tasks. The judges were graduate students of psychology enrolled in MA or Ph.D. programs who had at least moderate experience with cognitive psychology and problem solving. The judges were presented with a standard definition of insight and asked to evaluate the extent to which a given task complies with such a definition and requires insight problem solving. They were also asked to assess the familiarity of tasks, their logical and grammatical consistency, and their difficulty level on the 1–7 scale. In this way we tried to eliminate tasks the solutions to which would be known in advance to potential participants, tasks that would be unclear or ambiguous, as well as tasks that would be too easy or too difficult to solve. As a consequence, we obtained 31 tasks which did not raise any reservations from the judges’ side and whose difficulty level did not touch the extremes (items with 0 or 100% correct solutions were eliminated). As to the subjective ratings of difficulty, 11 tasks were judged quite difficult, difficult, or very difficult (on a scale of 5–7), 16 tasks were judged very easy, easy, or quite easy (on a scale of 1–3), whereas four tasks obtained the middle ratings of 4. This final pool of 31 items was used in the study proper (see Appendices 1 and 2 in Supplementary Material).

#### N-Back Task

Working memory updating was assessed with an experimental task called n-back (McElree, 2001; Owen et al., 2005). Participants were presented with two-digit numbers that appeared in the center of the screen and remained there either for 1800 ms or until the response. These stimuli were masked with random patterns of dots. Numbers filled in the 50 cm × 30 cm square with no apparent edges. After 500 ms of masking, another stimulus appeared on the screen. The task was to press the space key if and only if a given stimulus had been presented two (*n* = 2) or four (*n* = 4) items back. For instance, in the following stream of stimuli: 31 **56** 34 **56** 42 12 and so forth, the number 56 is repeated and a participant is supposed to recognize it as being presented two items back. Similarly, in the stream: **23** 45 34 56 **23** and so forth, the repeated number 23 has been presented previously four items back. There were six series of the n-back task, each consisting of 88 stimuli, out of which 16 would reappear in the proper position. Three series were prepared according to the easier rule of detection (*n* = 2), and three series required the more demanding rule (*n* = 4). The location of repeated stimuli (i.e., signals) within every series was prearranged in the quasi-random way and fixed for all participants. The procedure started with the *n* = 2 series, next it switched to the *n* = 4 series, back to the *n* = 2 series, and so on. Participants were instructed to update the contents of their WM in order to be able to know whether a given stimulus has been presented before at the predefined location. Updating is crucial for this task because of the fact that stimuli are constantly changing their position in the series. For instance, the stimulus that is now at the screen has the position *n* = 0 but after its disappearance it gains the position *n* = 1, next *n* = 2, and so forth. Participants were told which position was valid in every series: *n* = 2 or *n* = 4. They were also asked to ignore stimuli that did not match to the pattern kept in WM in the valid position.

Two indices of performance have been registered: the number of omissions and the number of false alarms. The former took place when a participant did not press the space key in spite of the fact that a given number had been repeated in the predefined position (*n* = 2 or *n* = 4). In contrast, false alarms were registered when a participant pressed the space key unnecessarily, that is, in response to a stimulus that had not been repeated at all. In this version of the n-back task we did not present participants with lures, that is, stimuli reappearing in wrong positions. In the *n* = 2 condition, they could be repeated too early (*n* = 1) or too late (*n* = 3). Such versions of the n-back task are particularly demanding for the cognitive control processes, as they required active inhibition of the prepotent albeit wrong response. Since we were primarily interested in WM updating rather than cognitive control, we decided to get rid of lures.

#### Raven’s Advanced Progressive Matrices

For intelligence assessment, we used Raven’s Advanced Progressive Matrices (RAPM, Raven et al., 1983) in the Polish adaptation by Jaworowska and Szustrowa (1991). This test consists of 10 introductory items and 36 main items, arranged according to the increasing difficulty. Each task requires the grasp of analogical relationships between abstract symbols. It allows good estimation of the general fluid intelligence, defined as the eduction of relations ability (Spearman, 1927). Due to time restrictions we administered only the main 36 items.

### Procedure

Participants first completed the computerized n-back test, which took about 15 min. There were short training sessions preceding the proper testing. Then, they completed Raven’s Matrices (25 min) and Insight Tasks (60 min). They were tested in a computer room equipped with separate cubicles.

## Results

Descriptive statistics are reported in **Table 1**. Both Raven’s Matrices and Insight Tasks were solved at the average level, without any indication of either floor or ceiling effects. Mean and median values were in the middle of the absolute range of results, which was between 0 and 36 in the case of Raven’s matrices and between 0 and 31 in the case of insight tasks. Both tests provided distribution not differing from normal, which was checked with the K–S test.

**Table 1:** Descriptive statistics for the initial sample (*N* = 89, upper lines) and the final sample (*N* = 81, lower lines).

	Mean	*SD*	Median	Minimum	Maximum
RAPM	19.81	3.04	20	12	26
	20.12	2.83	20	14	26
IT	20.63	3.77	21	11	28
	21.05	3.56	21	13	28
OM *n* = 2	0.34	0.09	0.35	0.07	0.51
	0.35	0.09	0.35	0.07	0.51
OM *n* = 4	0.37	0.12	0.38	0.08	0.60
	0.38	0.12	0.39	0.08	0.60
FA *n* = 2	0.04	0.05	0.03	0.00	0.24
	0.03	0.03	0.02	0.00	0.15
FA *n* = 4	0.11	0.08	0.10	0.00	0.38
	0.10	0.05	0.09	0.02	0.29

*RAPM, Raven’s Advanced Progressive Matrices; IT, Insight Tasks; OM, proportion of omissions; FA, proportion of false alarms; n = 2, n-back task, signals first appearing two items back; n = 4, n-back task, signals first appearing four items back.*

As to the computerized n-back task, the average proportion of omissions was 0.34 in the *n* = 2 condition and 0.37 in the *n* = 4 condition. Proportion of false alarms was 0.04 and 0.11, respectively in the *n* = 2 and *n* = 4 conditions. A closer look at the data suggested that eight participants, whose results did not differ statistically from the 50% chance level, lowered the average accuracy scores. Some of them also surpassed the “three sigma” criterion concerning the number of false alarms. These participants were excluded from further analyses on the basis of the argument that probably they did not follow the instruction or their cognitive skills were too low for the task’s requirements. In consequence, further analyses were applied to the sample of 81 people. Descriptive statistics concerning the final sample of 81 participants are presented in **Table 1**, too (the lower lines).

Both conditions of the n-back task differed in difficulty, which was checked with *t*-test for dependent samples. For the proportion of omissions, the difference between *n* = 2 and *n* = 4 conditions was significant at the *p* < 0.01 level, *F*(1,88) = 7.83. For the proportion of false alarms, this difference was significant at the *p* < 0.001 level, *F*(1,88) = 39.16. These differences were expected as confirmation of the theoretical validity of the n-back task.

In the next step, we checked reliability of the battery of Insight Tasks. The internal consistency measure (Cronbach’s α) for the whole battery was 0.62, which is a results usually interpreted as questionable (George and Mallery, 2003). A closer examination of items revealed that there were five tasks whose elimination increased the α index of the whole battery. After their removal, Cronbach’s α of the battery of the remaining 26 tasks was 0.71, which is an acceptable level of internal consistency (George and Mallery, 2003). All further analyses were therefore performed with the use of the 26-item version of the battery.

In order to verify our hypotheses, we computed correlation coefficients referring to the main variables of the study (**Table 2**). We found strong negative correlations between the number of correct responses in the battery of Insight Tasks and the proportion of omissions, both in the *n* = 2 and in the *n* = 4 conditions (*r* = -0.48 and *r* = -0.53, respectively). Relationships of the IT battery and the proportion of false alarms were much weaker, surpassing the level of statistical significance only in the relatively less demanding *n* = 2 condition. At the same time, we found even stronger relationships between the proportion of omissions and the Raven’s test scores (*r* = -0.54 and *r* = -0.58, respectively for the *n* = 2 and *n* = 4 conditions). All these correlations were negative, suggesting that the higher the scores in the IT or RAPM tests the better accuracy in the computerized n-back task. Strength of the observed relationships suggests that the ability to solve insight tasks shares about 25% of common variance with the ability to update the contents of WM, as long as the latter is measured with the proportion of omissions. The percentage of common variance was much smaller (about 5%) if WM task performance is assessed with the proportion of false alarms. By the way, these two aspects seem to be quite separate, since the proportion of omissions in the *n* = 2 condition was not correlated at all with the proportion of false alarms (*r* = 0.08), whereas in the *n* = 4 condition it was correlated negatively (*r* = -0.42, *p* < 0.001). These results suggest that omissions and false alarms, being both indicators of accuracy in the *n* = back task, refer to quite distinct aspects of WM functioning.

**Table 2:** Correlations matrix (*N* = 81).

	OM	OM	FA	FA	RAPM
	*n* = 2	*n* = 4	*n* = 2	*n* = 4	
OM *n* = 4	0.71^∗∗∗^				
FA n = 2	-0.08	-0.07			
FA n = 4	-0.30^∗∗^	-0.42^∗∗∗^	0.31^∗∗^		
RAPM	-0.54^∗∗∗^	-0.58^∗∗∗^	0.03	0.27^∗^	
IT	-0.48^∗∗∗^	-0.53^∗∗∗^	-0.23^∗^	0.16	0.41^∗∗∗^

*OM, proportion of omissions; FA, proportion of false alarms; n = 2, n-back task, signals first appearing two items back; n = 4, n-back task, signals first appearing four items back; RAPM, Raven’s Advanced Progressive Matrices; IT, Insight Tasks; ^∗^p < 0.05; ^∗∗^p < 0.01; ^∗∗∗^p < 0.001.*

It is also worth noticing (**Table 2**) that both ability measures were correlated positively at the moderate level (*r* = 0.41). If so, their correlations with n-back measures may be confounded by their mutual influence. Therefore, we computed partial correlation coefficients between n-back performance and the IT battery while controlling for RAPM. We also computed analogical correlations for RAPM, controlling for IT. We found that all significant correlations remained significant; however, their strength was a bit reduced. For IT, its partial correlations with OM were -0.36 and -0.47, respectively for the *n* = 2 and *n* = 4 conditions. For RAPM, respective partial correlations were -0.45 and -0.48. So, we can conclude that zero-order correlations reported in **Table 2** lost part of their strength with IT when RAPM was controlled for, and vice versa. However, the “pure” relationships, represented by partial correlations, were strong enough to justify a conclusion that the ability to solve insight tasks depends on WM updating, regardless of the confounding influence of general intelligence.

In the next step, we looked at the difficulty level of the tasks included in the IT battery. It appeared that eight of them obtained very high percentages of correct responses, thus not being able to differentiate the participants’ level of the ability to solve insight problems (see Appendix 3 in Supplementary Material). We suspected that, regardless of the fact that the whole battery did not reveal any indications of the ceiling effect, these eight items of reduced difficulty might contribute to lowering the values of correlation coefficients. Items without enough power to differentiate participants usually reduce variation and thus make correlation coefficients artificially low. Having removed eight items that appeared too easy, we checked for the correlation matrix again. The results are reported in **Table 3**.

**Table 3:** Zero-order and partial correlations between the reduced IT battery and n-back performance measures (*N* = 81).

		OM	OM	FA	FA	RAPM
		*n* = 2	*n* = 4	*n* = 2	*n* = 4	
IT	(Zero-order)	-0.45^∗∗∗^	-0.63^∗∗∗^	-0.24^∗^	0.14	0.37^∗∗∗^
IT	(Partial)	-0.31^∗^	-0.54^∗∗∗^	-0.28^∗^	0.04	–

*OM, proportion of omissions; FA, proportion of false alarms; n = 2, n-back task, signals first appearing two items back; n = 4, n-back task, signals first appearing four items back; RAPM, Raven’s Advanced Progressive Matrices; IT, Insight Tasks; ^∗^p < 0.05; ^∗∗∗^p < 0.001.*

As expected, the removal of eight easy tasks from the IT battery resulted in strengthening the correlations with n-back task measures. The correlation between IT and OM in the *n* = 4 condition seems particularly interesting because it increased from -0.53 (**Table 2**) to -0.63 (**Table 3**). Moreover, this correlation remained strong (-0.54) after partialling out the effects of Raven’s scores. It may then be concluded that the ability to solve insight problems shares about 30% of common variance with the ability to update the contents of WM, even if the influence of general mental ability is controlled for.

## Discussion

Most creativity researchers agree that insight problem solving requires restructuring of the problem representation. A marked lack of agreement concerns the mechanisms by which the restructuring occurs. Some authors propose that restructuring of the problem representation relies upon controlled search processes, suggesting an essential role for WM and planning in insight problem solving (MacGregor et al., 2001; Chein et al., 2010; Chein and Weisberg, 2014). Other researchers suggest that restructuring is achieved through the automatic spread of activation in long-term memory, assigning a limited role to WM processes (Ash and Wiley, 2006). The results of the present study reinforce the view that insight problem solving is related to WM involvement. Since we used the n-back task to investigate WM processes, we suggest that the function of WM updating is involved in insight problem solving. Although n-back, as many cognitive tasks, suffers from impurity, it is believed to engage at least two out of three postulated components of updating, namely *recognition* of already presented items and *substitution* of old items with new ones (Ecker et al., 2010). Interestingly, when comparing two indices of n-back, omission errors and false alarms, they both seemed to refer to distinct processes, with the former sharing much more common variance with insight problem solving then the latter (25 and 5% respectively). Finally, our results indicate that when controlled for IT task difficulty, a measure of updating function accounts for up to 30% of variance in the solution of insight problems, even when the influence of general mental ability is controlled for. To the best of our knowledge, this is the first study revealing the relationship between insight problem solving and WM updating function.

Let us analyze possible cognitive mechanisms responsible for the contribution of WM updating to insight problem solving. According to MacGregor et al. (2001), the difficulty in solving insight problems stems from the fact that a concrete goal is defined in abstract terms and therefore cannot be foreseen and used for progress monitoring. In such circumstances a successful strategy of problem solving depends critically on applying maximization and progress-monitoring heuristics. People may succeed only if, at each stage of the task, they try to choose a move that maximally reduces the difference between the current state and the sub-goal, and constantly monitor their progress against solution criteria (and not against the desired final goal, which is impossible). Consequently, the critical component of insight problem solving is the ability to envisage the situation that will be achieved after a series of steps. Thanks to this ability, the insightful move can be inspired not just by actual failure, but instead by the anticipation of failure. In the formal information-processing model proposed by MacGregor et al. (2001) this ability was implemented in a form of *lookahead* parameter. The suggestion that people use maximization and progress-monitoring heuristics with *lookahead* was supported empirically. MacGregor et al. (2001) revealed that human participants and computer models spend more time on solving the nine-dot problem, and its modifications varying in number of dots, if the problems are related to greater *lookahead*.

Building up on this work, Chein et al. (2010) and Chein and Weisberg (2014) operationalized *lookahead* mechanisms in terms of WM capacity. In the direct test of the role of *lookahead* in nine-dot performance, using an individual-differences approach, Chein et al. (2010) found that spatial WM capacity predicted the tendency to draw lines outside the configuration of dots, the solution of a hint-aided version of the problem, and shorter solution times of the nine-dot problem. In the subsequent study the authors (Chein and Weisberg, 2014) explored the contributions of WM and attention to the solution of compound remote associate problems (CRA). In the CRA, participants are required to find a solution word that is associated with three stimuli words provided (see also: Mednick, 1962, 1968). Particular solutions can be accompanied with the ‘aha’ experience, or not. Chein and Weisberg (2014) firstly divided the CRA problems into those whose solution was accompanied by a subjective feeling of insight on the basis of the participants’ self-reported insight ratings provided. Then, they examined the correlations between problem performance and measures of verbal WM and spatial WM capacity, as well as attentional control (by means of Stroop and antisaccade tasks). The results indicated that individual differences in both modality-specific and executive components of WM (i.e., those associated with the control of attention) explained a significant portion of variation in overall CRA problem solving and, most importantly, in the cases when problem solutions were accompanied by a subjective feeling of insight.

The results of the current study support the conclusion offered by Chein et al. (2010) and Chein and Weisberg (2014) by providing convergent evidence based on different methods, and extends their account by specifying further the nature of WM involvement in insight problem solving. In the present study WM updating was assessed with the n-back paradigm, as opposed to the OSPAN task used by Chein and Weisberg (2014). In OSPAN task the participants are required to perform a simple mathematical verification and then read a word or letter. After several such processing-and-storage presentations, a recall grid is presented, and people are required to indicate in serial order the words or letters they had seen previously. Operation span is determined on the basis of the highest number of words that can be recalled by the participant. In terms of construct validity, complex span tasks have been consistently shown to have stronger relations to memory tasks requiring information manipulation than to those demanding mainly rehearsal (e.g., Engle et al., 1999b). Therefore it is believed that WM span is a reliable predictor of complex cognitive behavior across domains, including problem solving, reasoning, and reading comprehension, because it is related to executive control (Engle et al., 1992; Conway et al., 2005).

Although n-back and OSPAN are similar in that they both require simultaneous storage and processing of the material, it is still a matter of debate whether they reflect primarily a single construct and whether findings from one of these tasks can be easily applied the to the other (Roberts and Gibson, 2002; Oberauer, 2005; Kane et al., 2007; Jaeggi et al., 2010; Redick et al., 2012). Several recent psychometric tests have shown a full range of results. In some cases n-back and WM span were shown to correlate weakly (Kane et al., 2007; Redick et al., 2012). For example, in the study by Kane et al. (2007) these tasks shared only 2–5% of their variance. Moreover, even though both tasks predicted variance in RAPM, they primarily did so independently, with less shared than unique predictive variance between them (see also: Oberauer, 2005; Redick et al., 2012). These results favor interpretation, according to which complex span tasks rely heavily on executive attention but do not involve updating, which, in contrast is strongly implicated in n-back performance.

In contrast, Schmiedek et al. (2009) obtained strong positive correlation between a latent factor measured by three complex span tasks and a latent factor represented by three different working-memory updating tasks (including figural n-back). Wilhelm et al. (2013) obtained a similar high construct overlap of recall-n-back and complex span, as well as a very strong relationship with the latent factor for updating. Noteworthy, these correlational studies measured WM through multiple indicators and evaluated their relationship through structural equation modeling (SEM). Hence, it was possible to overcome the shortcomings of other studies where WM and executive attention were tested with a single experimental paradigm, conflating variance due to individual differences in executive control with task-specific variance and resulting in null correlations (Wilhelm et al., 2013).

Moreover, the findings from our lab support the claim that n-back task reflects primarily the updating function of WM that is statistically identical to storage capacity (Chuderski and Nęcka, 2012; Chuderski et al., 2012). This conclusion was based on the observation that, in the above-mentioned studies, updating as measured by figural n-back task did not account for any amount of variance above and beyond the variance accounted for the scores reflecting maintenance of the pattern of a few items for several seconds (as measured by the array comparison task) or construction and maintenance of temporary bindings among perceptually available items (as measured by the two monitoring tasks). In fact, on the basis of these data the authors have questioned the existence of a distinct executive function of updating, amounting it to storage capacity (see also Wilhelm et al., 2013).

In sum, we believe that the relationship between n-back and insight problem solving, as revealed by the current investigation, concerns primarily the updating function of WM. Considering the results by Chuderski et al. (2012), updating function measured by n-back task used here amounts to storage capacity. Therefore it seems that insight problem solving ability may be crucially limited either by the number of items maintained in active memory or by the number of bindings which the individual is able to maintain in active memory (plus possible interaction of these factors). Presumably binding compromises storage and vice versa, in analogy to the relationship between primary memory and secondary memory proposed by Unsworth and Engle (2007b).

Obviously, on the basis of the current data we are unable to distinguish between the two aspects of updating: maintenance and binding (see: Chuderski et al., 2012). Speculating, one can assume that the maintenance component of the WM can be conceptualized in terms of the ‘*n*’ value in the n-back task: the higher the ‘*n*’ is, the more items have to be stored in memory simultaneously in order to generate correct match, and consequently, the more difficult the tasks becomes. Indeed, our results revealed that IT performance showed stronger correlation with the four-back condition of the current memory task than with the two-back condition. Interestingly, this relationship was observed only for omission errors. Arguably, such errors occur either due to a failure to maintain to-be-remembered item in active memory, or due to a failure to generate proper binding within a series of items. The mechanism of false alarms seem much more elusive, as it amounts to “seeing” an item or a binding which is simply not there, possibly due to some source of proactive interference that emerges across task trials. In this case, proactive interference drove the association between insight problem solving and WM updating as measured by false alarms. Alternatively, false alarms may have reflected an individual’s impulsive strategy to overreact (Saunders et al., 2008).

The idea offered here that insight problems solving depends heavily on maintenance and binding processes corresponds clearly to the binding hypothesis of WM capacity offered by Oberauer et al. (2007), and Wilhelm et al. (2013) in the context of fluid intelligence: “WM is important for reasoning because reasoning requires the construction and manipulation of representations of novel structures. The limited capacity of WM arises from interference between bindings, which effectively limits the complexity of new structural representations, and thereby constrains reasoning ability” (Wilhelm et al., 2013, p. 4).

The current study has certain limitations. In methodological terms, the most important limitation is that we did not study insight directly, and more importantly, we did not assess its critical aspects: impasse and restructuring (see Chein and Weisberg, 2014). Instead, we used a correlational design allowing us only to relate such a global measure as the IT index to WM updating performance. Although the IT task items used here were carefully selected and judged, it is still possible that IT index used here conflates many factors. One important factor overlooked in the present study relates to the characteristics of the problems included into the IT task. It has been suggested that different types of insight tasks require different forms of restructuring (Ohlsson, 1984a,b, 1992; Weisberg, 1995), e.g., the requirement for figure-ground type reversals, the degree of misdirection involved, the need to redefine spatial assumptions, and so on (see Cunningham et al., 2009). Clearly some insight problems are more difficult to solve than others, and this difficulty is affected by characteristics of the restructuring processes required (Cunningham et al., 2009). Problem characteristics may also mediate the relationship between insight problem solving and WM. Ash and Wiley (2006) found that high WM capacity (as measured by WM span tasks) predicted an individual’s ability to successfully solve problems that involve both the initial search phase and the restructuring phase. However, individual differences in WM capacity did not predict success on problems that isolated the restructuring phase only. The current data supports these claims indirectly, that is, when several IT items were excluded from the analysis due to their insufficient discriminating power, the correlation between IT and WM updating increased. This finding suggests that the relationship between insight problem solving and WM updating depends on the level of task difficulty, which, in turn, may be related to restructuring characteristics of the task.

In summary, our results point to the conclusion that insight problem solving depends on WM updating, i.e., maintenance of items in WM and rapid binding of the incoming information with current sub-goals maintained in WM. WM updating, conceptualized as the combination of maintenance and binding, probably allows to form a new representations of a problem space. Investigation of insight problem solving in terms of updating function with an inclusion of restructuring characteristics may be the promising direction for future research on individual differences in insight problem solving.

## Author Contributions

All authors listed, have made substantial, direct and intellectual contribution to the work, and approved it for publication.

## Conflict of Interest Statement

The authors declare that the research was conducted in the absence of any commercial or financial relationships that could be construed as a potential conflict of interest.

## References

[B1] Ansburg P. I. (2000). Individual differences in problem solving via insight. *Curr. Psychol.* 19 143–146. 10.1016/j.actpsy.2015.02.004

[B2] Ash I. K. Wiley J. (2006). The nature of restructuring in insight: an individual-differences approach. *Psychon. Bull. Rev.* 13 66–73. 10.3758/BF03193814 16724770

[B3] Baddeley A. D. (2002). Is working memory still working? *Eur. Psychol.* 7 85–97. 10.1027//1016-9040.7.2.85

[B4] Baddeley A. D. Hitch G. J. (1974). “Working memory,” in *Recent Advances in Learning and Motivation* Vol. 8 ed. Bower G. A. (New York, NY: Academic Press), 47–90.

[B5] Beaty R. E. Silvia P. J. Nusbaum E. C. Jauk E. Benedek M. (2014). The roles of associative and executive processes in creative cognition. *Mem. Cogn.* 42 1186–1197. 10.3758/s13421-014-0428-8 24898118

[B6] Benedek M. Jauk E. Fink A. Koschutnig K. Reishofer G. Ebner F. (2014). To create or to recall? Neural mechanisms underlying the generation of creative new ideas. *Neuroimage* 88 125–133. 10.1016/j.neuroimage.2013.11.021 PMC3991848 24269573

[B7] Benedek M. Neubauer A. C. (2013). Revisiting Mednick’s model on creativity-related differences in associative hierarchies. Evidence for a common path to uncommon thought. *J. Creat. Behav.* 47 273–289. 10.1002/jocb.35 24532853 PMC3924568

[B8] Chein J. M. Weisberg R. W. (2014). Working memory and insight in verbal problems: analysis of compound remote associates. *Mem. Cogn.* 42 67–83. 10.3758/s13421-013-0343-4 23864281

[B9] Chein J. M. Weisberg R. W. Streeter N. L. Kwok S. (2010). Working memory and insight in the none-dot problem. *Mem. Cogn.* 38 883–892. 10.3758/MC.38.7.883 20921101

[B10] Chu Y. MacGregor J. N. (2011). Human performance on insight problem solving: a review. *J. Probl. Solv.* 3 119–150. 10.7771/1932-6246.1094

[B11] Chuderski A. Nęcka E. (2012). The contribution of working memory to fluid reasoning: capacity, control, or both? *J. Exp. Psychol. Learn. Mem. Cogn.* 38 1689–1710. 10.1037/a0028465 22612170

[B12] Chuderski A. Taraday M. Nęcka E. Smoleñ T. (2012). Storage capacity explains fluid intelligence but executive control does not. *Intelligence* 40 278–295. 10.1016/j.intell.2012.02.010

[B13] Conway A. R. A. Kane M. J. Bunting M. F. Hambrick D. Z. Wilhelm O. Engle R. W. (2005). Working memory span tasks: a methodological review and user’s guide. *Psychon. Bull. Rev.* 12 769–786. 10.3758/BF03196772 16523997

[B14] Cowan N. (2001). The magical number 4 in short-term memory: a reconsideration of mental storage capacity. *Behav. Brain Sci.* 24 87–114. 10.1017/S0140525X01003922 11515286

[B15] Cowan N. (2010). The magical mystery four: how is working memory capacity limited, and why? *Curr. Dir. Psychol. Sci.* 19 51–57. 10.1177/0963721409359277 20445769 PMC2864034

[B16] Csikszentmihalyi M. (1996). *Creativity: Flow and the Psychology of Discovery and Invention.* New York, NY: HarperCollins Publishers.

[B17] Cunningham J. B. MacGregor J. N. Gibb J. Haar J. (2009). Categories of insight and their correlates: an exploration of relationships among classic-type insight problems, rebus puzzles, remote associates and esoteric analogies. *J. Creat. Behav.* 43 262–280. 10.1002/j.2162-6057.2009.tb01318.x

[B18] Davidson J. E. (1995). “The suddenness of insight,” in *The Nature of Insight*, eds Sternberg R. J. Davidson J. E. (Cambridge, MA: MIT Press),125–155.

[B19] De Dreu C. K. W. Nijstad B. A. Baas M. Wolsink I. Roskes M. (2012). Working memory benefits creative insight, musical improvisation, and original ideation through maintained task-focused attention. *Pers. Soc. Psychol. Bull.* 38 656–669. 10.1177/0146167211435795 22301457

[B20] DeCaro M. S. Van Stockum C. A. Wieth M. B. (2015). When higher working memory capacity hinders insight. *J. Exp. Psychol. Learn. Mem. Cogn.* 42 39–49. 10.1037/xlm0000152 26120772

[B21] Dominowski R. L. Dallob P. (1995). “Insight and problem solving,” in *The Nature of Insight*, eds Sternberg R. J. Davidson J. E. (Cambridge, MA: MIT Press), 157–196.

[B22] Dow G. T. Mayer R. E. (2004). Teaching students to solve insight problems: evidence for domain specificity in creativity training. *Creat. Res. J.* 16 389–398. 10.1207/s15326934crj1604_2

[B23] Dunbar K. (1995). “How scientists really reason: scientific reasoning in real-world laboratories,” in *The Nature of Insight*, eds Sternberg R. J. Davidson J. E. (Cambridge, MA: MIT Press), 365–395.

[B24] Ecker U. K. H. Lewandowsky S. Oberauer K. Chee A. E. H. (2010). The components of working memory updating: an experimental decomposition and individual differences. *J. Exp. Psychol. Learn. Mem. Cogn.* 36 170–189. 10.1037/a0017891 20053053

[B25] Engle R. W. (2002). Working memory capacity as executive attention. *Curr. Dir. Psychol. Sci.* 11 19–23. 10.1111/1467-8721.00160

[B26] Engle R. W. Cantor J. Carullo J. J. (1992). Individual differences in working memory and comprehension: a test of four hypotheses. *J. Exp. Psychol. Learn. Mem. Cogn.* 18 972–992. 1402719 10.1037//0278-7393.18.5.972

[B27] Engle R. W. Kane M. J. Tuholski S. W. (1999a). “Individual differences in working memory capacity and what they tell us about controlled attention, general fluid intelligence, and functions of the prefrontal cortex,” in *Models of Working Memory: Mechanisms of Active Maintenance and Executive Control*, eds Miyake A. Shah P. (Cambridge: Cambridge University Press),102–134.

[B28] Engle R. W. Tuholski S. W. Laughlin J. E. Conway A. R. (1999b). Working memory, short-term memory, and general fluid intelligence: a latent-variable approach. *J. Exp. Psychol. Gen.* 128 309–331. 10.1037/0096-3445.128.3.309 10513398

[B29] Finke R. A. (1996). Imagery, creativity, and emergent structure. *Conscious Cogn.* 5 381–393. 10.1006/ccog.1996.0024 8906409

[B30] Finke R. A. Ward T. B. Smith S. M. (1992). *Creative Cognition: Theory, Research, and Applications.* London: The MIT Press.

[B31] Friedman N. P. Miyake A. Corley R. P. Young S. E. DeFries J. C. Hewitt J. K. (2006). Not all executive functions are related to intelligence. *Psychol. Sci.* 17 172–179. 10.1111/j.1467-9280.2006.01681.x 16466426

[B32] George D. Mallery P. (2003). *SPSS for Windows Step by Step: A Simple Guide and Reference. 11.0 Update*, 4th Edn. Boston, MA: Allyn & Bacon.

[B33] Gick M. L. Lockhart R. S. (1995). “Cognitive and affective components of insight,” in *The Nature of Insight*, eds Sternberg R. J. Davidson J. E. (Cambridge, MA: The MIT Press), 197–228.

[B34] Gilhooly K. J. (2005). “Working memory and planning,” in *The Cognitive Psychology of Planning*, eds Morris R. Ward G. (London: Routledge),71–88.

[B35] Gilhooly K. J. Fioratou E. (2009). Executive functions in insight versus non-insight problem solving: an individual differences approach. *Think. Reason.* 15 355–376. 10.1080/13546780903178615

[B36] Gilhooly K. J. Murphy P. (2005). Differentiating insight from non-insight problems. *Think. Reason.* 11 279–302. 10.1080/13546780442000187

[B37] Guilford J. P. (1950). Creativity. *Am. Psychol.* 5 444–454. 10.1037/h0063487 14771441

[B38] Guilford J. P. (1967). *The Nature of Human Intelligence*. New York, NY: McGraw-Hill.

[B39] Isaak M. I. Just M. A. (1995). “Constraints on thinking and invention,” in *The Nature of Insight*, eds Stermberg R. S. Davidson J. E. (Campridge, MA: The MIT Press), 281–325.

[B40] Jaeggi S. M. Buschkuehl M. Perrig W. J. Meier B. (2010). The concurrent validity of the N -back task as a working memory measure. *Memory* 18 394–412. 10.1080/09658211003702171 20408039

[B41] Jaworowska A. Szustrowa T. (1991). *Podrkęcznik do testu Martyc Ravena [Manual for the Raven’s Matrices test].* Warszawa: Pracownia Testów Psychologicznych PTP.

[B42] Jung R. E. Wertz C. J. Meadows C. A. Ryman S. G. Vakhtin A. A. Flores R. A. (2015). Quantity yields quality when it comes to creativity: a brain and behavioral test of the equal-odds rule. *Front. Psychol.* 25:864 10.3389/fpsyg.2015.00864 PMC4479710 26161075

[B43] Kane M. J. Conway A. R. A. Miura T. K. Colflesh G. J. H. (2007). Working memory, attention control, and the n-back task: a question of construct validity. *J. Exp. Psychol. Learn. Mem. Cogn.* 33 615–622. 17470009 10.1037/0278-7393.33.3.615

[B44] Langley P. Jones R. (1988). “A computational model of scientific insight,” in *The Nature of Creativity. Contemporary Psychological Perspectives*, ed. Sternberg R. J. (Cambridge: Cambridge University Press), 177–201.

[B45] Lavric A. Forstmeier S. Rippon G. (2000). Differences in working memory involvement in analytical and creative tasks: an ERP study. *NeuroReport* 11 1613–1618. 10.1097/00001756-200006050-00004 10852211

[B46] MacGregor J. N. Ormerod T. C. Chronicle E. P. (2001). Information processing and insight: a process model of performance on the nine-dot and related problems. *J. Exp. Psychol. Learn. Mem. Cogn.* 27 176–201. 10.1037/0278-7393.27.1.176 11204097

[B47] McElree B. (2001). Working memory and focal attention. *J. Exp. Psychol. Learn. Mem. Cogn.* 27 817–835. 11394682 PMC3077110

[B48] Mednick S. A. (1962). The associative basis of the creative process. *Psychol. Rev.* 69 220–232. 10.1037/h0048850 14472013

[B49] Mednick S. A. (1968). The remote associates test. *J. Creat. Behav.* 2 213–214.

[B50] Metcalfe J. Wiebe D. (1987). Intuition in insight and non-insight problem solving. *Mem. Cogn.* 15 238–246. 10.3758/BF03197722 3600264

[B51] Miyake A. Friedman N. P. Emerson M. J. Witzki A. H. Howerter A. Wager T. D. (2000). The unity and diversity of executive functions and their contributions to complex “frontal lobe” tasks: a latent variable analyses. *Cogn. Psychol.* 41 49–100. 10.1006/cogp.1999.0734 10945922

[B52] Morris N. Jones D. M. (1990). Memory updating in working memory: the role of the central executive. *Br. J. Psychol.* 81 111–121. 10.1111/j.2044-8295.1990.tb02349.x

[B53] Murray M. A. Byrne R. M. J. (2005). “Attention and working memory in insight problem solving,” in *Proceedings of the XXVII Annual Conference of the Cognitive Science Society*, eds Bara B. G. Barsalou L. Bucciarelli M. (Mahwah, NJ: Erlbaum), 1571–1575.

[B54] Nęcka E. (1999). Creativity and attention. *Pol. Psychol. Bull.* 30 85–97.

[B55] Oberauer K. (2005). Binding and inhibition in working memory: individual and age differences in short-term recognition. *J. Exp. Psychol. Gen.* 134 368–387. 10.1037/0096-3445.134.3.368 16131269

[B56] Oberauer K. (2009). Design for a working memory. *Psychol. Learn. Motiv.* 51 45–100. 10.1016/S0079-7421(09)51002-X

[B57] Oberauer K. Süß H.-M. Wilhelm O. Sander R. (2007). “Individual differences in working memory capacity and reasoning ability,” in *Variation in Working Memory*, eds Conway A. Jarrold C. Kane M. Miyake A. Towse J. (Oxford: Oxford University Press), 49–75.

[B58] Ohlsson S. (1984a). Restructuring revisited: summary and critique of the Gestalt theory of problem solving. *Scand. J. Psychol.* 25 65–78.

[B59] Ohlsson S. (1984b). Restructuring revisited: an information processing theory of restructuring and insight. *Scand. J. Psychol.* 25 117–129. 10.1111/j.1467-9450.1984.tb01005.x

[B60] Ohlsson S. (1992). “Information-processing explanations of insight and related phenomena,” in *Advances in the Psychology of Thinking* Vol. 1 eds Keane M. T. Gilhooly K. J. (London: Harvester Wheatsheaf), 1–44.

[B61] Ormerod T. C. Chronicle E. P. MacGregor J. N. (2006). “Asymmetrical analogical transfer in insight problem solving,” in *Proceedings of the 28th Annual Conference of the Cognitive Science Society*, ed. Sun R. (Mahwah, NJ: Lawrence Erlbaum Associates, Inc), 1899–1904.

[B62] Osborn A. F. (1953). *Applied Imagination.* New York, NY: Charles Scribner’s Sons.

[B63] Owen A. M. McMillan K. M. Laird A. R. Bullmore E. (2005). N-back working memory paradigm: a meta-analysis of normative functional neuroimaging studies. *Hum. Brain Mapp.* 25 46–59. 10.1002/hbm.20131 15846822 PMC6871745

[B64] Palmiero M. Cardi V. Belardinelli M. O. (2011). The role of vividness of visual mental imagery on different dimensions of creativity. *Creat. Res. J.* 23 372–375. 10.1080/10400419.2011.621857

[B65] Parnes S. J. Meadow A. (1959). Effects of “brainstorming” instructions on creative problem solving by trained and untrained subjects. *J. Educ. Psychol.* 50 171–176. 10.1037/h0047223

[B66] Paulewicz B. Chuderski A. Nęcka E. (2007). “Insight problem solving, fluid intelligence, and executive control: a structural equation modeling approach,” in *Proceedings of the 2nd European Cognitive Science Conference*, eds Vosniadou S. Kayser D. Protopapas A. (Hove: Laurence Erlbaum), 586–591.

[B67] Raven J. C. Court J. H. Raven J. (1983). *Manual for Raven’s Progressive Matrices and Vocabulary Scales (Section 4, Advanced Progressive Matrices).* London: H. K. Lewis.

[B68] Redick T. S. Broadway J. M. Meier M. E. Kuriakose P. S. Unsworth N. Kane M. J. (2012). Measuring working memory capacity with automated complex span tasks. *Eur. J. Psychol. Assess.* 28 164–171. 10.1027/1015-5759/a000123

[B69] Roberts R. Gibson E. (2002). Individual differences in sentence memory. *J. Psychol. Res.* 31 573–598. 10.1023/A:1021213004302 12599915

[B70] Saunders B. Farag N. Vincent A. S. Collins F. L. Sorocco K. H. Lovallo W. R. (2008). Impulsive errors on a Go-NoGo reaction time task: disinhibitory traits in relation to a family history of alcoholism. *Alcohol. Clin. Exp. Res.* 32 888–894. 10.1111/j.1530-0277.2008.00648.x 18373725 PMC2836919

[B71] Scheerer M. (1963). Problem solving. *Sci. Am.* 208 118–128. 10.1038/scientificamerican0463-118 13986996

[B72] Schmiedek F. Hildebrandt A. Lövdén M. Wilhelm O. Lindenberger U. (2009). Complex span versus updating tasks of working memory: the gap is not that deep. *J. Exp. Psychol. Learn. Mem. Cogn.* 35 1089–1096. 10.1037/a0015730 19586272

[B73] Schooler J. W. Ohlsson S. Brooks K. (1993). Thoughts beyond words: when language overshadows insight. *J. Exp. Psychol. Gen.* 122 166–183. 10.1037/0096-3445.122.2.166

[B74] Seifert C. M. Meyer D. E. Davidson N. Patalano A. L. Yaniv I. (1995). “Demystification of cognitive insight: opportunistic assimilation and the prepared-mind perspective,” in *The Nature of Insight*, eds Sternberg R. J. Davidson J. E. (Cambridge, MA: MIT Press), 65–124.

[B75] Simon H. A. (1977). *Models of Discovery.* Dordrecht: Reidel.

[B76] Simon H. A. Langley P. Bradshaw G. (1981). Scientific discovery as problem solving. *Synthese* 47 1–27. 10.1007/BF01064262

[B77] Simonton D. K. (1988). *Scientific Genius: A Psychology of Science.* New York, NY: Cambridge University Press.

[B78] Simonton D. K. (1997). Creative productivity: a predictive and explanatory model of career trajectories and landmarks. *Psychol. Rev.* 104 66–89. 10.1037/0033-295X.104.1.66

[B79] Sio U. N. Ormerod T. C. (2009). Does incubation enhance problem solving? A meta-analysis. *Psychol. Bull.* 135 94–120. 10.1037/a0014212 19210055

[B80] Smith S. M. Ward T. B. Finke R. A. (eds) (1995). *The Creative Cognition Approach.* Cambridge, MA: MIT Press.

[B81] Spearman C. (1927). *The Abilities of Man.* London: Macmillan.

[B82] Unsworth N. Engle R. W. (2007a). On the division of short-term and working memory: an examination of simple and complex span and their relation to higher order abilities. *Psychol. Bull.* 133 1038–1066. 10.1037/0033-2909.133.6.1038 17967093

[B83] Unsworth N. Engle R. W. (2007b). The nature of individual differences in working memory capacity: active maintenance in primary memory and controlled search from secondary memory. *Psychol. Rev.* 114 104–132. 10.1037/0033-295X.114.1.104 17227183

[B84] Van Stockum C. DeCaro M. S. (2013). “The path less taken: when working memory capacity constrains insight,” in *Proceedings of the 35th Annual Conference of the Cognitive Science Society*, eds Knauff M. Pauen M. Sebanz N. Wachsmuth I. (Berlin: Cognitive Science Society), 3633–3638.

[B85] Wallach M. A. Kogan N. (1965). *Modes of Thinking in Young Children.* New York, NY: Holt.

[B86] Ward T. B. (1994). Structured imagination: the role of category structure in exemplar generation. *Cogn. Psychol.* 27 1–40. 10.1006/cogp.1994.1010

[B87] Weisberg R. W. (1995). “Prolegomena to theories of insight in problem solving: a taxonomy of problems,” in *The Nature of Insight*, eds Sternberg R. J. Davidson J. E. (Cambridge, MA: MIT Press), 157–196.

[B88] Wertheimer M. (1945). *Productive Thinking.* New York, NY: Harper and Row.

[B89] Wilhelm O. Hildebrandt A. Oberauer K. (2013). What is working memory capacity, and how can we measure it? *Front. Psychol.* 4:433 10.3389/fpsyg.2013.00433 PMC3721021 23898309

[B90] Yeh Y.-C. Tsai J.-L. Hsu W.-C. Lin C. F. (2014). A model of how working memory capacity influences insight problem solving in situations with multiple visual representations: an eye-tracking analysis. *Think. Skills Creat.* 13 153–167. 10.1016/j.tsc.2014.04.003

