# Supplementary material for: Insightful Imagery is Related to Working Memory Updating
Source: Front Psychol. 2016 Feb 29;7:137. doi: 10.3389/fpsyg.2016.00137 (PMC4770025; doi:10.3389/fpsyg.2016.00137)
Supplement: Supplementary file 1 [file Data_Sheet_1.PDF]

<u>Appendix 1: Insight Ability Test</u>

Instruction:

This test consists of a sample of several dozen problems of varying degree of complexity. Try to solve as many of them as you can. If you cannot solve a particular problem, skip it and try to unravel the next one. You have 60 minutes to complete the test.

Task 1

The matter takes place in an old monastery, home to circa 20 monks. They spend their days praying ardently. They have nothing accept for a simple bed in a modest room, one chair and a window, no mirrors or commodities. Every night God comes to them and talks to each one of them separately. One night, He tells them that there is one monk suffering from a shameful disease. Its symptom is the presence of a blue spot on the front of the sick person. The monk has to work out that he is ill and leave the monastery. The monks meet up only during the meals. Attention, no other monk may indicate anything or mention his disease to him and he has no possibility to see his face in the mirror. How is he supposed to get to know that he is the one who contracted the disease?

Response:

____

____

____

Task 2

A man went to the ZOO and saw giraffes and ostriches. Altogether, they had 30 pairs of eyes and 44 legs. How many animals did the man see?

Response:

Task 3

Join the 4 dots up with 2 continuous straight lines without lifting your pencil from the paper.

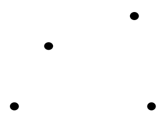

Task 4

There are 1801 people living in a small village located in Bieszczady, and only 5% of them have a phone number registered under their name. 100 names was randomly selected from a telephone directory of the village. Please, try to estimate how many peoples from this sample would belong to the group whose phones are not registered.

Response:

Task 5

A fast electric train runs at 150 km/hour north. The wind blows 30 km/hour east. Which way does the smoke generated by the train go?

Response:

____

____

____

Task 6

A father and his son had a serious accident. The man was killed and the son was rushed to the emergency room. Upon arrival, the attending doctor looked at the child and said: „I can't operate on him because he is my son”. How is that possible?

Response:

____

____

____

Task 7

A woman unwrapped a lump sugar cube and put it into a coffee. Sugar did not dissolve however. How is that possible?

Response:

____

Task 8

There are 7 sisters in the Kowalski family, and each sister has one brother. How many men belong to the family, including Mr. Kowalski?

Response:

Task 9

A girl has four 3-link chains. She wants to join all of them into a big chain. It costs 2 zloty to break a link, and 3 zloty to weld it again. The girl has only 15 zloty. How can she make it?

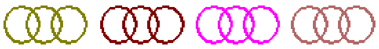

Response:

Task 10

How can you divide this figure into four parts that have the same size and shape.

Depict the response.

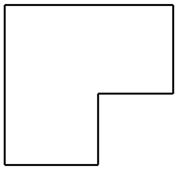

Task 11

We have 10 sacks of identically looking coins and an electronic scale with a precision of 1 g. Each sack has 10 coins in it. One sack contains fake coins, whilst the remaining nine sacks contain authentic coins. An authentic coin weighs 10 g, whilst a fake coin weights 9 g. How can you find out in which sack the fake coins are, if only one measurement is allowed?.

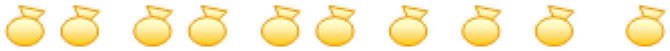

Response:

Task 12

Last week our basketball team won 73- 49. None of the men scored more than 1 point. How is that possible?

Response:

Task 13

Bartek is a nephew of Maciek’s father. Maciek is a nephew of Bartek’s father. Who are Maciek and Bartek for each other?

Response:

____

____

____

__

Task 14

Tomek never tells the truth. Marek never tells lies. One of them said: „The other said that I am Marek”. Who said that?

Response:

____

____

____

Task 15

A globetrotter is walking around in mountains. It becomes dark and snowing. Freezing cold he gets to a shelter, where he finds two oil lamps, a candle and several pieces of wood for burning stove. He has only one match. What does he light first?

Response:

____

Task 16

Using only six toothpicks form four equilateral triangle.

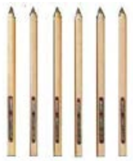

Depict the response:

Task 17

On the picture below there are ten dots forming an arrow that points to a certain direction. Now moving only three dots make an arrow that points into *the opposite direction*.

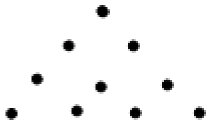

Depict the response:

Task 18

Three couples of friends went together to a party. The first woman was wearing a red dress, the second woman was wearing a green dress, and the third one was wearing a blue dress. Each men matched his partner in terms of a color of their clothing. At some point, when a man in red was dancing with a woman in blue, one of the women said: „Isn't it funny that none of us is dancing with a partner who is wearing the same color?". Which man was dancing with the woman in red?

Response:

____

____

____

Task 19

Three women – Joanna, Danuta and Sandra – have three kids in total – Dominik, Karolina and Daniel. Dominik likes to play with Danuta's son. Sandra occasionally looks after Joanna's kids. Who is Karolina's mother?

Response:

____

____

____

Task 20

In a pitch dark room in a sock drawer there are black and brown socks, with a ratio 4 to 5. How many socks must you take out to ensure that you have at least one color matching pair?

Response:

____

____

____

Task 21

A new company doubles its sale every month. At the end of December it amounted to 10 thousands zloty. When did the value of the sale amount to 5 thousands zloty?

Response:

____

____

____

Task 22

A legendary runner was so fast that his friends used to say that he can turn off the light and jump into the bed before it becomes dark. One day the runner proved that he can actually do that. How did he do that?

Response:

____

____

____

Task 23

An archaeologist declared that he found a coin dated 21 B.C. Another archeologist immediately claimed it was a lie. Why?

Response:

____

____

____

Task 24

A brother with his sister and a husband with his wife found four coins. Each of them took one coin, and one was spare. How is that possible?

Response:

____

____

____

Task 25

There are two identical wallets. The first one contains 120 g of 1 euro coins made of a pure gold, and the second one contains 60 g of 2 euro coins made also of a pure gold. Which wallet is worth more?

Response:

____

____

____

Task 26

A boat has a ladder made up of 12 steps. The distance between steps is of 20 cm. During low tide, six steps are submerged. How many steps will be under water when the tide comes in increasing the level of water 60 cm?

Response:

Task 27

Please, put 10 dots so that they form five rows with four dots in each row.

○ ○ ○ ○ ○ ○ ○ ○ ○ ○

Depict the response:

Task 28

Tomek went for a walk without an umbrella. He neither wear a cap, nor hide from the rain. Still, not a single hair on his head got wet. How could it happen?

Response:

Task 29

How can you stand behind another person, whilst this person is standing right behind you?

Response:

Task 30

In the light of the Catholic Church rules, is it possible for a man to marry a sister of his widow?

Response:

Task 31

You fly in a plane over an ocean. You fling out 5 kilos of stones and 5 kilos of feathers through a window. What will fall down on the gound first?

Response:
