# Supplementary material for: Insightful Imagery is Related to Working Memory Updating
Source: Front Psychol. 2016 Feb 29;7:137. doi: 10.3389/fpsyg.2016.00137 (PMC4770025; doi:10.3389/fpsyg.2016.00137)
Supplement: Supplementary file 2 [file Data_Sheet_2.PDF]

Appendix 2. Insight Tasks – solutions

Task 1

Response: A monk who is sick, has a blue spot on his front. He has no mirror but he has got his eyes. When the monks meet up for a meal, he can look at their faces and if he sees no spot on the faces of the other monks, he is contracted and has to leave the monastery.

Task 2

Response: Fifteen (30/2)

Task 3

Response:

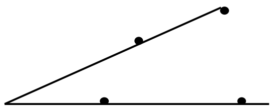

Task 4

Response: None.

Task 5

Response: There is no smoke – this is an electric train.

Task 6

Response: The doctor was a mother.

Task 7

Response: A woman put a lump sugar cube into a grained or instant coffee.

Task 8

Response: Two (Mr Kowalski and his son).

Task 9

Response: Open three links of one chain and use them to join the rest.

Task 10

Response:

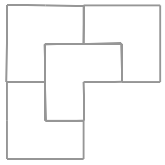

Task 11

Response: One should take one coin from the first sack, two coins from the second sack, three coins from the third sack, and so on. If all of them would weight 10 g than we have (100 + 90 +80 + 70 + 60 + 50 + 40 + 30 +20 + 10)= 550g. If the scale shows 543 g it means that fake coins are in the sevents sack, if the scale shows 548 it means that the fake coins are in the second sack.

Task 12

Response: It was a female basketball team.

Task 13

Response: They were cousins.

Task 14

Response: Marek (he always tells the truth, so he meant that Tomek - who always tells lie - said he was Marek)

Task 15

Response: A match.

Task 16

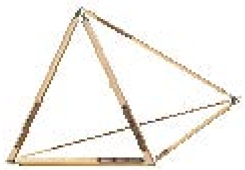

Response:

Task 17

Response:

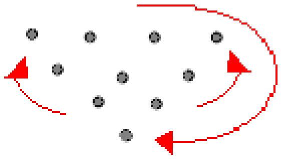

Task 18

Response: A man in green. A man in red was dancing with a woman in blue. A man in green had two options: a woman in green or in red. Since none of the pairs was colour-matched, he could only be dancing with a women in red.

Task 19

Response: Joanna

Task 20

Response: 3 socks.

Task 21

Response: At the end of November.

Task 22

Response: He did it during a day.

Task 23

Response: It was indicated by the B.C. (Before Christ) inscription.

Task 24

Response: There were three people: either a brother and a sister with her husband, or a sister and a brother with his wife.

Task 25

Response: 120g of coins made off pure gold worth more than 60g coins made of the same material.

Task 26

Response: Number of steps under water does not change when the tide comes in.

Task 27

Response:

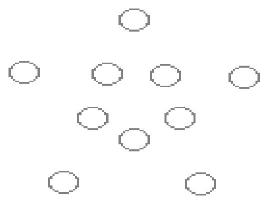

Task 28

Response: It wasn't raining.

Task 29

Response: Standing back-to-back with each other.

Task 30

Response: No, because he would be already dead.

Task 31

Response: None, because you fly over an ocean.
