# Supplementary material for: Insightful Imagery is Related to Working Memory Updating
Source: Front Psychol. 2016 Feb 29;7:137. doi: 10.3389/fpsyg.2016.00137 (PMC4770025; doi:10.3389/fpsyg.2016.00137)
Supplement: Supplementary file 3 [file Data_Sheet_3.PDF]

Appendix 3: Insight Tasks – basic information

Item #	Alpha after removal	Proportion correct	Removed from final version?
1	higher	47	YES (rel.)
2	lower	91	YES (discr.)
3	higher	56	YES (rel.)
4	higher	82	YES (rel. + discr.)
5	lower	33	NO
6	lower	58	NO
7	lower	81	NO
8	lower	65	NO
9	lower	48	NO
10	lower	21	NO
11	lower	43	NO
12	lower	82	YES (discr.)
13	higher	52	YES (rel.)
14	lower	70	NO
15	higher	88	YES (rel. + discr.)
16	lower	43	NO
17	lower	85	YES (discr.)
18	lower	75	NO
19	lower	84	YES (discr.)
20	lower	70	NO

21	lower	71	NO
22	lower	56	NO
23	lower	96	YES (discr.)
24	lower	62	NO
25	lower	64	NO
26	lower	51	NO
27	lower	48	NO
28	lower	81	YES (discr.)
29	lower	75	NO
30	lower	87	YES (discr.)
31	lower	99	YES (discr.)

discr. - removed from the short version because of low discriminability; rel. - removed from the short version because of low reliability.
